# Riluzole-Loaded Nanostructured Lipid Carriers for Hyperproliferative Skin Diseases

**DOI:** 10.3390/ijms24098053

**Published:** 2023-04-29

**Authors:** Xavier Llorente, Gerard Esteruelas, Lorena Bonilla, Mariana Garnica Agudelo, Ingrid Filgaira, Daniel Lopez-Ramajo, Ruoyi C Gong, Concepció Soler, Marta Espina, Maria Luisa García, Joan Manils, Montserrat Pujol, Elena Sánchez-López

**Affiliations:** 1 Department of Pharmacy, Pharmaceutical Technology and Physical Chemistry, Faculty of Pharmacy, University of Barcelona, 08028 Barcelona, Spain; xllorelo8@alumnes.ub.edu (X.L.); gesteruelas@ub.edu (G.E.); lbonilla95@ub.edu (L.B.); mgarniag26@alumnes.ub.edu (M.G.A.); m.espina@ub.edu (M.E.); marisagarcia@ub.edu (M.L.G.); 2 Institute of Nanoscience and Nanotechnology (IN2UB), University of Barcelona, 08028 Barcelona, Spain; 3 Departament de Patologia i Terapèutica Experimental and Hospital Universitari de Bellvitge-Bellvitge Institute for Biomedical Research, 08907 Barcelona, Spain; ifilgaira@ub.edu (I.F.); dlopezramajo@ub.edu (D.L.-R.); rg3271@columbia.edu (R.C.G.); concepciosoler@ub.edu (C.S.); joanmanils@ub.edu (J.M.); 4 Serra Húnter Programme, Immunology Unit, Department of Pathology and Experimental Therapy, School of Medicine, Universitat de Barcelona, Feixa Llarga s/n, 08907 L’Hospitalet de Llobregat, Spain; 5 Unit of Synthesis and Biomedical Applications of Peptides, IQAC-CSIC, 08034 Barcelona, Spain

**Keywords:** nanostructured lipid carriers, dermal administration, psoriasis, Riluzole, lipid nanoparticles, drug delivery system

## Abstract

Nanocarriers, and especially nanostructured lipid carriers (NLC), represent one of the most effective systems for topical drug administration. NLCs are biodegradable, biocompatible and provide a prolonged drug release. The glutamate release inhibitor Riluzole (RLZ) is a drug currently used for amyotrophic lateral sclerosis (ALS), with anti-proliferative effects potentially beneficial for diseases with excessive cell turnover. However, RLZ possesses low water solubility and high light-sensibility. We present here optimized NLCs loaded with RLZ (RLZ-NLCs) as a potential topical treatment. RLZ-NLCs were prepared by the hot-pressure homogenization method using active essential oils as liquid lipids, and optimized using the design of experiments approach. RLZ-NLCs were developed obtaining optimal properties for dermal application (mean size below 200 nm, negative surface charge and high RLZ entrapment efficacy). In vitro release study demonstrates that RLZ-NLCs allow the successful delivery of RLZ in a sustained manner. Moreover, RLZ-NLCs are not angiogenic and are able to inhibit keratinocyte cell proliferation. Hence, a NLCs delivery system loading RLZ in combination with natural essential oils constitutes a promising strategy against keratinocyte hyperproliferative conditions.

## 1. Introduction

The skin, being the largest outermost organ of the human body, interacts with many external stimuli and factors, such as temperature, mechanical, chemical and biological threats [1]. Such interactions can destabilize the epidermal function, triggering a response to adapt and protect itself, to maintain the barrier function [2]. A common skin response is inflammation, which is also triggered by internal physiological altered factors. In susceptible individuals, this response can lead to the development of inflammatory skin conditions, the incidence of which has increased more than 46 % in the last decades [3,4,5].

Chronic inflammatory dermatoses such as psoriasis are complex diseases with no cure, affecting more than 125 million people worldwide [3]. They are multifactorial diseases with the involvement of both genetic background and environmental factors [4]. For instance, the pathophysiology of psoriasis comprises excessive proliferation and abnormal differentiation of keratinocyte, and infiltration of immune cells, resulting in symmetrical erythematous and scaly plaques [2]. Hyperproliferative skin condition patients are treated depending on the extent of the disease; treatment ranges from topical to systemic biological therapies [5,6,7]. Although highly effective, some patients do not respond to biological therapies, and the ones that respond may become resistant. In addition, biologics have numerous unwanted side effects and require life-long periodic expensive inj ections [8]. Hence, there is a pressing need to expand treatment possibilities for the management of these disorders. When exploring novel pharmacological candidates, a currently used drug with unexplored bioactivity can be a much-needed alternative.

Riluzole (RLZ) is a drug belonging to the benzothiazoles group, and it is currently approved for amyotrophic lateral sclerosis due to its neuroprotective and anticonvulsive activities [9]. RLZ has been advocated to possess the potential to reduce angiogenesis [10], inhibit cancer cell proliferation [11], and exert antioxidant effects by the inhibition of protein kinase C [12] and phospholipase A [13]. Given the pathophysiological mechanisms of hyperproliferative skin conditions, these effects might be potentially beneficial to treat them. In addition, it has been predicted that RLZ may inhibit xCT (Cystine/glutamate transporter), a ferroptosis-associated gene highly expressed in psoriasis [14]. Of note, keratinocytes express components of the glutamate signaling pathway [15]. Taking these facts into account, we hypothesize that RLZ could be an effective treatment for keratinocyte-based proliferative skin diseases, such as psoriasis. However, RLZ is sensitive to light and possesses low aqueous solubility, thus affecting its bioavailability and leading to complex side effects, like angioedema or hepatitis, among others [16].

In order to improve RLZ administration, avoiding its degradation and obtaining a sustained release, nanostructured carriers have been postulated as suitable alternatives for drug encapsulation. For several years, nanotechnological applications have led to important and innovative breakthroughs, continuously being explored in areas such as medicine and industrial fields. Lipid nanoparticles, a type of nanostructured system, constitute a colloidal delivery system composed of a lipid matrix and an appropriate stabilizing surfactant [17]. Lipid nanoparticles are biocompatible, offer protection of encapsulated molecules against degradation, as well as a prolonged drug release [18]. Among lipid nanoparticles, nanostructured lipid carriers (NLCs) are composed of solid and liquid lipids which allow a larger space for drug accommodation, avoiding the on-storage drug expulsion [19]. In this area, lipids such as beeswax, lavender oil and peppermint oil are known for their wound healing, anti-inflammatory and analgesic properties [20,21,22]. Therefore, a combination of the drug loading capacity of NLCs, the intrinsic activity of the lipids and the entrapment of RLZ may have a great potential for the treatment of compromised skin barrier function disorders, and may constitute a good candidate for topical application in skin inflammatory conditions such as psoriasis [23,24].

In this work, a novel formulation based on NLCs and loading RLZ (RLZ-NLCs) has been developed and optimized. RLZ-NLCs have been physicochemically characterized and in vitro RLZ release has been studied. In addition, anti-angiogenic potential has been assessed in ovo and anti-proliferative effects have been assessed in keratinocytes, showing RLZ-NLCs potential to ameliorate hyperproliferative skin conditions.

## 2. Results

### 2.1. Optimization of RLZ-NLCs

**Figure 1:**
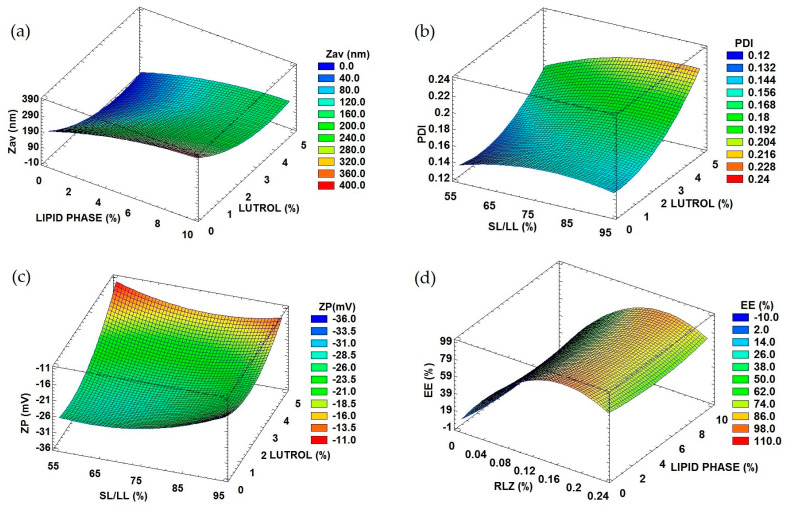
Response surface of optimized RLZ-NLCs. (**a**) Lutrol^®^ F68 and lipid phase influence on Z_av_. (**b**) Lutrol^®^ F68 and lipid phase influence on PI. (**c**) Solid lipid/liquid lipid [SL/LL] and Lutrol^®^ F68 influence on ZP. (**d**) Lipid phase and RLZ influence on EE.

To optimize RLZ-NLCs formulation, a design of experiment (DoE) approach was performed (Table 1). In Figure 1a, the surface response showed that average size (Z_av_) was significantly affected by the concentration of surfactant, Lutrol^®^ F68 ([LUT]), as well as the lipid phase amount, in a significantly direct manner. This can also be observed in the Pareto diagram (Figure S1a).

The polydispersity index (PI) was significantly affected by [LUT] and by the ratio of solid vs liquid lipid (SL/LL), as shown in Figure 1b and the Pareto diagram (Figure S1b). Despite this, PI did not exceed values of 0.2, corresponding to a monomodal population.

Regarding the zeta potential (ZP) (Figure 1c, Figure S1c), a measure of the electrical potential at the slipping plane of the nanoparticles, it was significantly affected by the ratio of SL/LL, the surfactant and the drug amount. The ratio SL/LL was the most influencing variable, where the higher the values, the higher the ZP (up to −30 mV). Moreover, at high surfactant values, high ZP were also obtained.

Regarding the entrapment efficiency (EE), meaning the percentage of drug incorporated into the nanocarriers with respect to the initial drug quantity, values ranged from 50 to 85%, as shown in Figure 1d and the Pareto diagram (Figure S1d). This dependent variable was significantly influenced by RLZ concentration in a directly proportional manner.

Taking these trends into account, the optimized RLZ-NLCs formulation was extrapolated and it is shown in Table 2 [16]. Optimized RLZ-NLCs possess a Z_av_ below 200 nm, which might allow them to cross through the stratum corneum and obtain dermal drug delivery, something that might not be achieved with major sizes [25]. ZP values around −30 mV should provide a stable system by preventing the possible aggregation of RLZ-NLCs [26]. Regarding PI, desirable values under 0.2 which correspond to monomodal systems were obtained [27]. Concerning the EE, the optimized formulation showed a suitable EE of 87.2%.

### 2.2. Interaction Studies

**Figure 2:**
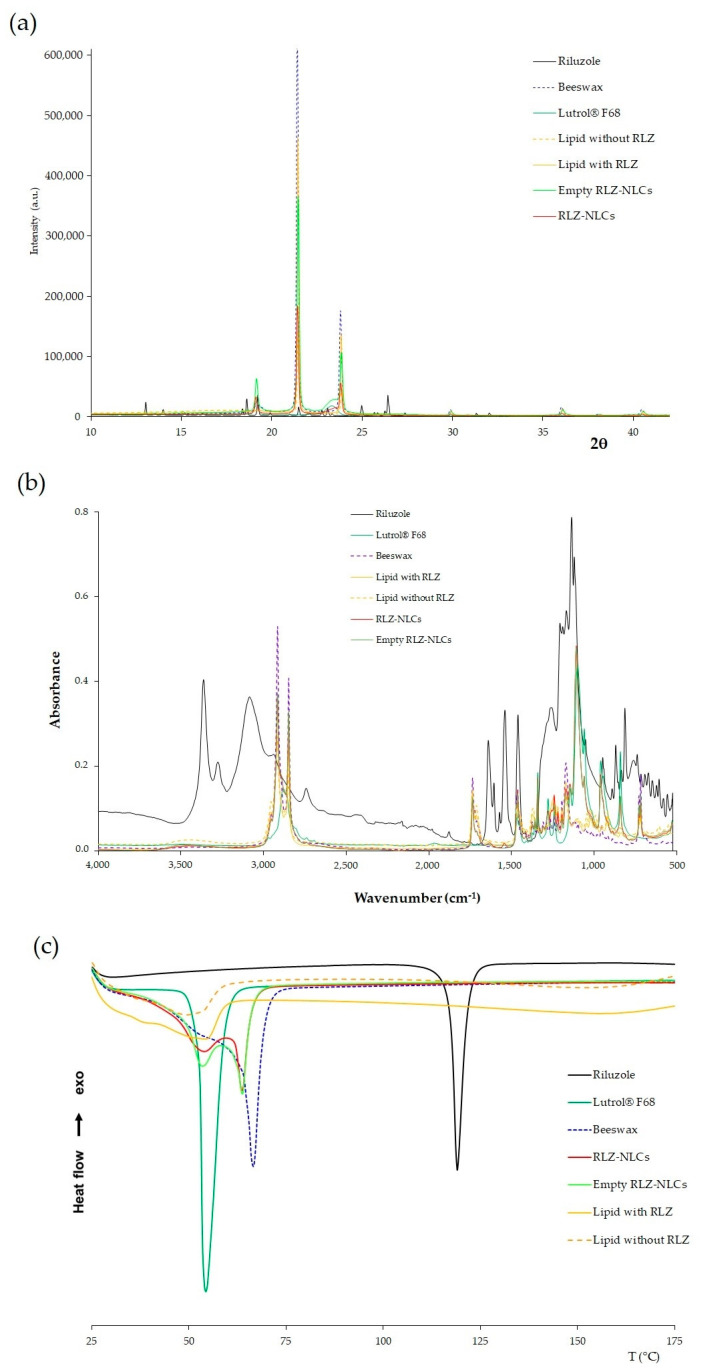
Interaction studies for RLZ-NLCs optimized formulation and their components. (**a**) XRD diffraction patterns. (**b**) FTIR profile. (**c**) DSC thermal profile.

The interaction between RLZ-NLCs and their components was studied (Figure 2). First, the X-ray diffraction (XRD) profile (Figure 2a) of RLZ showed sharp peaks at different diffraction angles (13.0°, 14.0° 18.6°, 19.2°, 21.8°, 23.1°, 25.0°, 26.4°, 31.3°, and 32.0°) due to its crystalline structure [28]. These peaks were absent in the diffractograms of the RLZ-NLCs and in the drug-lipid mixture (SL and LL melt with RLZ). Instead, their diffractograms exhibited peaks attributed to beeswax, with peaks at 19.2°, 21.5°, 23.8°, 29.9°, 36.0° and 40.6°. Nonetheless, the peaks were less intense and wider compared to those of the beeswax alone. These same peaks were observed for the empty NLCs and the lipid melted without RLZ.

Fourier-transform infrared (FTIR) spectroscopy analysis (Figure 2b) showed two main bands for the RLZ, the wider one at 3077 cm^−1^ and the narrower at 3358 cm^−1^, which illustrates the stretching of the primary amine group (N-H) [29]. Moreover, several peaks corresponding to RLZ benzothiazole aromatic ring can be observed between 813 and 871 cm^−1^ corresponding to C-H bending, and between 1458 and 1630 cm^−1^ corresponding to the C=C and C=N stretching [28]. Additionally, the C-F_3_ group can be found in absorption peak range between 1230 and 1410 cm^−1^ [16]. Regarding the LUT spectrum, the absorption band observed at 1094 cm^−1^ was attributed to the characteristic C-O stretching vibrations of the repeated polyethylene glycol units of LUT. Moreover, the major peak displayed at 2860 cm^−1^ represents C-H symmetric stretching vibrations [30]. Beeswax spectrum presented characteristic peaks at 1720, 1456 and 1163 cm^−1^, corresponding to C=O stretching, C-C stretching and C=O stretching vibrations, respectively [31]. In addition, two peaks were observed at 2924 and 2844 cm^−1^, corresponding to C-H asymmetric and symmetric stretching vibrations, respectively.

Comparing the melted lipid with and without RLZ, the absorption bands match in both cases with the same peaks, and these peaks were comparable with those of the beeswax spectrum, while no absorption bands ascribed to RLZ were visible. Similar results were obtained for NLC with and without RLZ, with bands corresponding to beeswax in both cases, in addition to the characteristic peaks of Lutrol^®^ F68. These results were expected due to the high content of lipids compared to RLZ, and indicate that RLZ was successfully entrapped in the nanoparticles matrix, since drug absorption bands were not observed in the nanoparticles spectrum.

The RLZ thermogram showed a single sharp peak characteristic of crystalline compounds (Figure 2c), corresponding to its melting temperature (T_m_) at 119.3 °C [29]. Lutrol^®^ F68 also showed a single endothermic sharp peak at 54.5 °C, indicating its melting point. Beeswax displayed a melting onset temperature around 41.7 °C, with a melting temperature (T_m_) at 66.7 °C, which agrees with the reported melting point [32]. Concerning the melted lipids without and with RLZ, the peaks observed for both mixtures were broader in comparison with the beeswax thermogram, with a shift to lower temperatures with an onset at 25 °C, and in the T_m_ observed at 49 °C and 55 °C, respectively. These differences are attributed to the interaction of the solid lipid with the liquid lipids and the surfactant [33]. NLCs with and without RLZ exhibit the same T_m (onset)_ at 32 °C, but both formulations presented two peaks due to two melting events. The first one at 54 °C corresponds to the melting of the surfactant present on the surface of the nanoparticles. This event may lead to loss of structure of the nanoformulations. The second peak was observed at 63.8 °C, indicating the melting of the lipid bulk. Finally, the RLZ peak was not observed in the RLZ-NLCs thermogram. These results suggest that the drug was successfully solubilized in the lipid matrix and encapsulated in the nanoparticles [33].

### 2.3. In Vitro Release Profile

**Figure 3:**
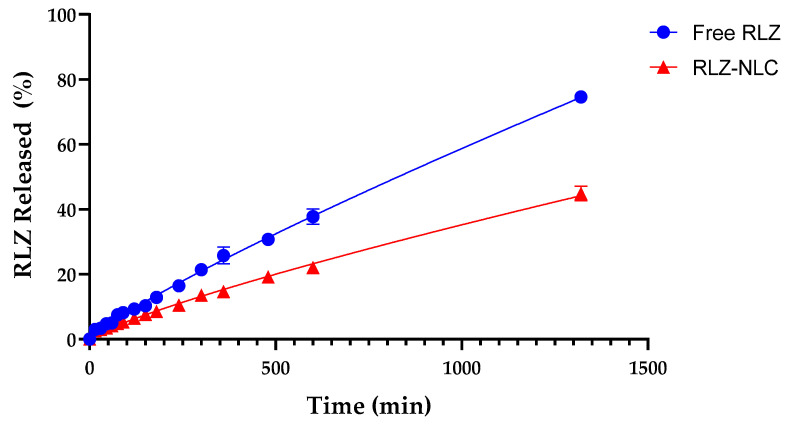
In vitro drug release of free RLZ and RLZ-NLCs in 5% Tween^®^ 80 solution at pH 7.4.

The in vitro release profile of RLZ against RLZ-NLCs was assessed. Free RLZ, dissolved in 5% Tween^®^ 80, showed a constant release with 75 % of the drug released during 24 h (Figure 3). RLZ-NLCs showed an initial burst effect followed by a sustained release, reaching 45% of RLZ released after 24 h. Of note, free RLZ experimented a release slower than expected, probably due to the use of Tween^®^ 80 as a dialysis medium. As reported by other authors, this surfactant has hydrophobic regions that have strong adsorption properties for small hydrophobic drugs, such as RLZ, delaying the release of the drug into the medium [34].

Different mathematical models to study drug release kinetics were tested in order to find the best fitting model. The Korsmeyer-Peppas model (Table 3) was the best fit for RLZ-NLCs (r^2^ = 0.9934), with a drug release rate constant (K) value of 0.1589. Normally, this model is used to describe polymeric systems, but it had been reported by other authors that this kinetic model could also apply for drug release from NLC systems [35]. Moreover, the release exponent for RLZ-NLCs (*n* = 0.8161) indicates that the release mechanism is dominated by anomalous diffusion (as 0.45 *< n <* 0.89), which implies that the release mechanism combines a diffusion process and lipid erosion [36].

### 2.4. Short-Term Stability

**Figure 4:**
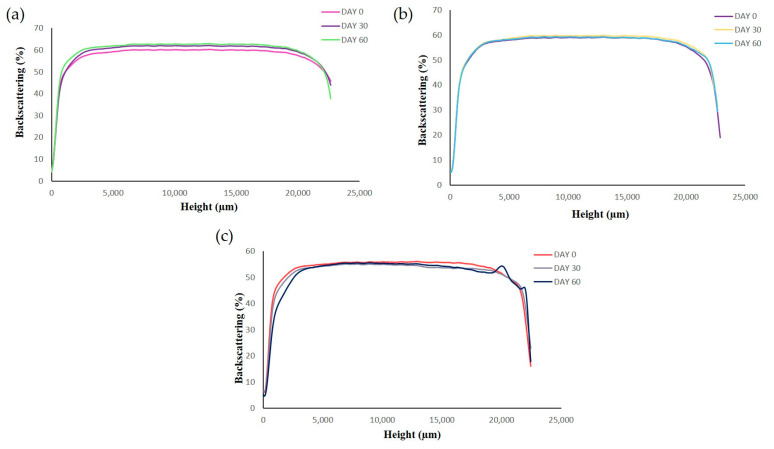
Backscattering profiles of RLZ-NLCs at different storage temperatures; (**a**) 4 °C, (**b**) 25°C and (**c**) 38 °C.

Short-term stability of RLZ-NLCs was studied at 3 temperatures (4, 25 and 38 °C) by means of backscattering profile (BS) and physicochemical analysis of the samples. BS indicated that after 60 days stored at 4 °C (Figure 4a) and 25 °C (Figure 4b), the optimized RLZ-NLCs were stable and differences in destabilization below 10% (creaming, sedimentation, flocculation, and coalescence) were registered. At storage temperatures of 38 °C (Figure 4c), results showed an initial instability process with differences still below 10 %.

Physicochemical analysis (Table 4) showed that the best storage temperature was 4 °C. At both 4 and 25 °C, particle size slightly decreased at day 60, although values below 200 nm were maintained. Concerning PI results, values were maintained under 0.2, indicating a narrow distribution, and ZP showed a slight decrease during the first month at all temperatures. Physicochemical results for storage at 25 and 38 °C showed major changes compared to 4 °C. Despite 4 °C being the best storage temperature, NLCs are thermodynamically unstable systems but they can be freeze-dried in order to increase their storage stability.

### 2.5. In Vitro Irritation Assay

**Figure 5:**
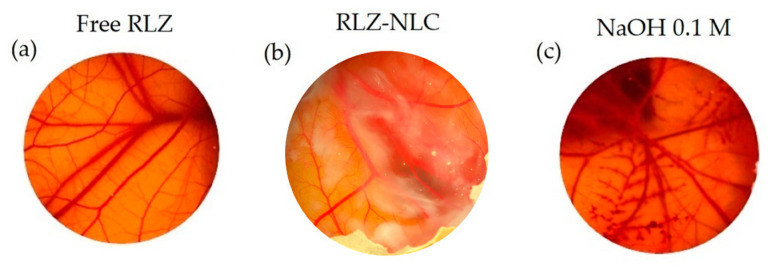
HET-CAM irritation test. (**a**) Free-RLZ, (**b**) RLZ-NLCs and (**c**) NaOH 0.1 M.

Due to the fact that dermal products usually tend to contact with the ocular surface, ocular tolerance of these drug delivery systems should be assessed for safety purposes. Therefore, HET-CAM (Hen’s Egg Chorioallantoic membrane test) qualitative test and HET-CAM quantitative test (HET-CAM TBS) were performed. As the HET-CAM results show in Figure 5c, the positive control of NaOH 0.1 M effectively generated a severe irritation of CAM (vasoconstriction, coagulation, and haemorrhage were observed); whereas the negative control was non-irritant. Free RLZ (Figure 5a) produced a slight vasoconstriction, being classified as weakly irritant, as previously reported [16,37]. Nevertheless, RLZ-NLCs (Figure 5b) did not produce any irritation phenomena being classified as non-irritant.

**Figure 6:**
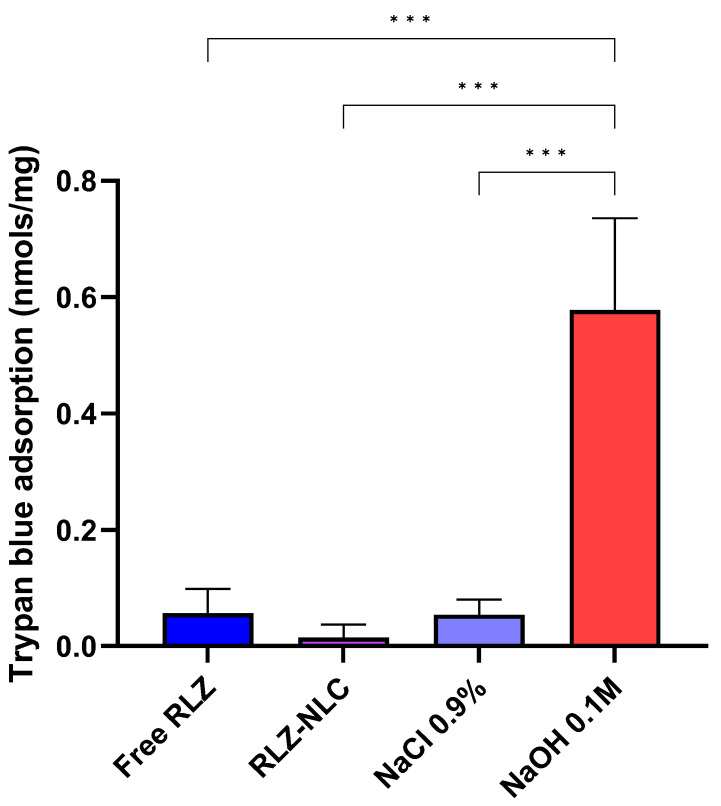
HET-CAM TBS results. Differences between groups were analyzed by one-way ANOVA. (*** *p* < 0.001).

The HET-CAM TBS assay results showed significant differences (*p* < 0.001) between NaOH 0.1 M and the other groups (Figure 6).

Our HET-CAM results are in correlation with CAM-TBS, both being classified as non-irritant.

### 2.6. Angiogenesis Capacity

**Figure 7:**
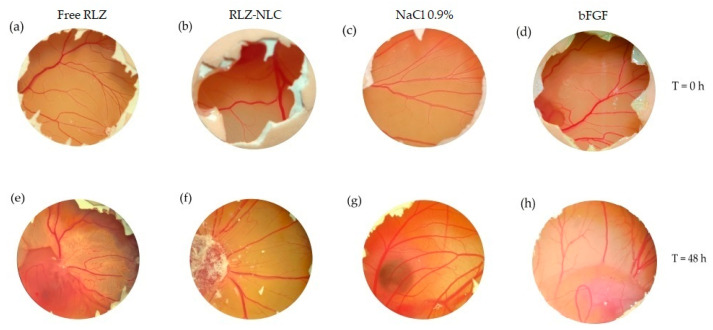
RLZ and RLZ-NLCs angiogenic capacity. Time = 0 h: (**a**) free RLZ, (**b**) RLZ-NLCs, (**c**) NaCl 0.9% and (**d**) bFGF. Time 48 h: (**e**) free RLZ, (**f**) RLZ-NLCs, (**g**) NaCl 0.9% and (**h**) bFGF.

The angiogenic potential of free RLZ, RLZ-NLCs and empty NLC formulations was studied by microscopical observation immediately after the addition of the products and after 48 h. After observation, the membrane was fixed with paraformaldehyde. NaCl 0.9 % and bFGF were used as controls (Figure 7).

**Figure 8:**
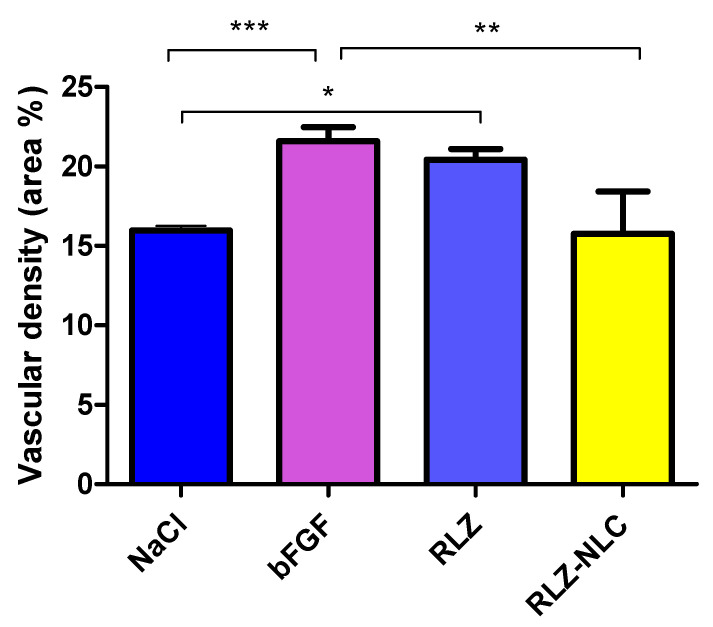
Measurement of the vascular area (%) of the extracted membranes after 48 h of stimulation with each compound. Differences between groups analyzed by one-way ANOVA; (* *p* < 0.05; ** *p* < 0.01; ***p < 0.005).

Membranes were extracted 24 h after the addition of paraformaldehyde, and imaged to be subsequently measured (Figure 8). Data analysis showed significant differences in vascular density between the control groups and the free RLZ and RLZ-NLCs.

Results show that free RLZ demonstrated angiogenic capacity, since significant differences (*p* < 0.05) against the negative control (NaCl) were obtained, but no significance was obtained against bFGF. On the other hand, RLZ-NLCs showed significant differences (*p* < 0.01) against bFGF. This difference between free RLZ and RLZ-NLCs may be due to RLZ sustained release from the NLCs, which after 24 h are able to release around 40% of the drug. Therefore, NLCs with prolonged RLZ release are able to overcome RLZ angiogenic capacity.

### 2.7. Inhibition of Proliferation

**Figure 9:**
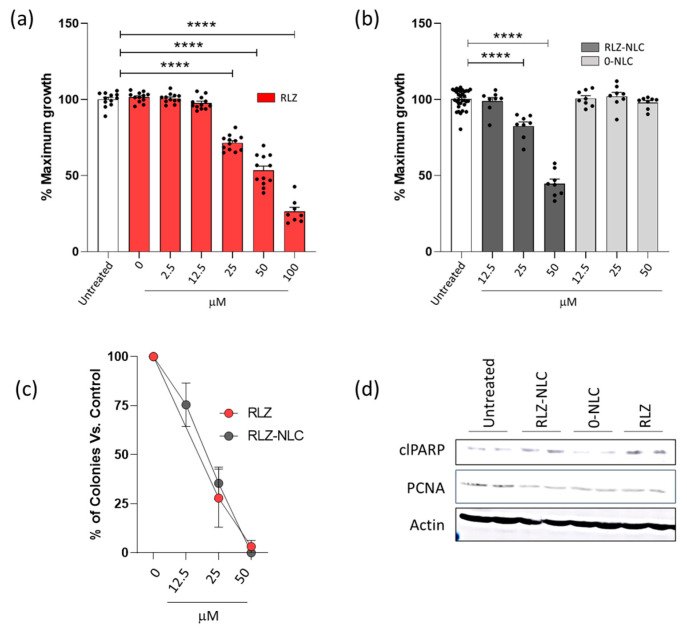
Effects of RLZ and RLZ-NLCs on HaCaT cells. (**a**) HaCaT cells were seeded in 96 well plates; 24 h after, cells were treated with increasing concentrations of Riluzole or vehicle (DMSO); and 48 h after, cell proliferation was determined as described in Materials and Methods. Graphs show cell growth percentage related to non-treated cells. (**b**) Cells were left untreated or treated with RLZ-NLCs and 0-NLC, and 48 h after, cell growth was assessed as in (**a**). (**c**) HaCaT cells were seeded in 6 well plates and treated as in (**b**); 10 days after, plates were fixed and stained to assess colony formation capacity. Each condition is referred to its controls (DMSO for RLZ, and 0-NLC for RLZ-NLCs). (**d**) Cells were treated with 25 μM RLZ, RLZ-NLCs or 0-NLC; 48 h after, stimulation protein was extracted. Lysates were resolved in SDS-PAGE and detection of proteins was performed by western blot with ECL. Data is expressed as mean ± SEM from one representative experiment out of three independent experiments (**a**,**b**,**d**) and a pool of 3 independent experiments. Differences between groups analyzed by one-way ANOVA; **** *p* < 0.0001.

RLZ ability to block glutamate release has been shown to be an effective tool in preventing proliferation of cells from different types of cancer [11]. To assess this feature in keratinocytes, the human keratinocyte cell line HaCaT was exposed to RLZ and RLZ-NLCs. A range of RLZ concentrations was added to the cells and growth was assessed using AlamarBlue reagent after 48 h. As previously shown for other cell types, RLZ significantly inhibited cell proliferation in a dose dependent manner, and at concentrations higher than 25 µM significant differences were obtained (*p* < 0.0001) (Figure 9a). Afterwards, the ability of NLC to retain the anti-proliferative property of RLZ on keratinocytes was assessed. RLZ-NLCs inhibited HaCaT keratinocyte growth akin to free RLZ (Figure 9b); of note, empty NLCs (0-NLC) did not show any anti-hyperproliferative effects. In addition, reduction of colony formation was similar when cells were treated with either free RLZ or RLZ-NLCs (Figure 9c), thus confirming that RLZ prolonged release from RLZ-NLCs does not diminish RLZ anti-hyperproliferative activity.

Induction of apoptosis after treatment with RLZ has been described in hepatocellular carcinoma [38] and nasopharyngeal carcinoma [39] cell lines. We tested whether RLZ and RLZ-NLCs promoted apoptosis of HaCaT cells. Western blot analysis of protein extracts from cells treated with RLZ or RLZ-NLCs showed higher levels of cleaved PARP (a marker of apoptosis) and decreased levels of PCNA (a proliferation marker) (Figure 9c). Taken together, these results show that growth inhibition properties of RLZ on keratinocytes, preventing proliferation and promoting cell death are preserved throughout the NLC complexation and synthesis.

## 3. Discussion

Aiming at a formulation for the dermal application of RLZ-NLCs [40], beeswax was chosen as the solid lipid, lavender and peppermint essential oils mixture as the liquid lipids, and Lutrol^®^ F68 as surfactant (a key component due to its reported beneficial effects in NLCs preparation [41]). Lavender and peppermint essential oils had been proposed for application of dermal preparations [23], and resulted in a promising combination for RLZ solubilization during RLZ-NLCs preparation.

After performing the DoE for RLZ-NLCs, RLZ and the ratio SL/LL did not demonstrate a statistical influence on Z_av_, probably due to the fact that during NLCs preparation the oil content is in a melted state [42]. We observed that the surfactant concentration was inversely proportional to the Z_av_. This might be related to an insufficient amount of surfactant to cover the nanoparticles at low concentrations; therefore, their stability decreases, which results in coalescence and aggregation of the nanoparticles [43]. Also, it has been reported that Z_av_ tends to be influenced by lipid and surfactant concentrations due to its straight relation with viscosity factor, which increases if lipid concentrations are near or above 10 %, resulting in an average size increase [44]. According to PI results, the surfactant concentration highly influences PI, which may be explained by recent reports showing that Lutrol^®^ F68 creates a stabilizer layer in the nanoparticle surface, leading to PI values around 0.2 [44,45]. Regarding ZP decrease, this can be due to the fact that non-ionic surfactants, such as Lutrol^®^ F68, usually tend, at high concentrations, to modify the charge to a more neutral one, resulting in a favorable stabilizer layer without compromising the stability [16,45]. The ZP values above 30 mV correspond to suitable ZP, as it allows stabilizing the NLCs dispersion while preventing the possible aggregation due to the repulsive interaction among particles [46]. Lastly, higher RLZ amounts increased the EE, as NLCs possess high loading capacity due to the liquid lipids added, and also because RLZ lipophilic and low solubility nature favors the interaction with the lipid matrix [47,48]. Therefore, the DoE led to an optimized formulation of RLZ-NLCs with values adequate for skin application purposes (Z_av_ around 200 nm; PI < 0.2; ZP ± 30 mV and EE ≥ 70 %) [49,50].

Regarding the physicochemical interactions, in XRD, RLZ peaks demonstrated the crystalline nature of the drug [33], and the RLZ-NLC spectrum showed that RLZ was successfully encapsulated inside the lipid matrix [51]. FTIR analysis showed that RLZ absorption bands are not present in the melted lipid spectrum nor in the RLZ-NLC spectrum, confirming that RLZ was solubilized in the melted lipid and successfully entrapped in the lipid matrix of the NLCs [33]. Moreover, no covalent bonds seemed to be formed in RLZ-NLCs [16]. DSC thermal analysis of RLZ-NLCs showed two T_m_ at 54 and 63.8 °C that can be explained as the melting of the surfactant at the surface of the nanoparticles and the melting of the lipid bulk, respectively. In addition, RLZ melting peak was not observed in RLZ-NLCs, indicating that the drug was encapsulated in a molecular dispersed state inside the NLCs, and confirming complete solubilization [33].

Free RLZ showed a delay on its release profile due to the fact that it was assessed in solution with Tween^®^ 80. This surfactant has strong adsorption properties for hydrophobic drugs like RLZ, which would explain the slower release of free RLZ [34]. In our optimized RLZ-NLCs formulation, a significant change in RLZ release was observed compared to free RLZ, confirming that these nanoparticles provide a sustained release of the encapsulated drug. These results are in agreement with previous reports showing a slow degradation of the NLCs lipid matrix, which correlates with a slower, more controlled drug release [52]. Importantly, slow and sustained drug liberation is key to prolong the effects of NLCs components over time, a feature not always achieved with current psoriasis drugs [7,53]. Inability to produce prolonged drug action results in partial relief, limiting the recovery of the epidermal barrier, which is essential to prevent the more complex dysregulated differentiation and proliferation of keratinocytes [54,55].

Regarding the HET-CAM test, RLZ-NLCs did not cause any irritation phenomena [56,57]. In the CAM-TBS assay, free RLZ and RLZ-NLCs trypan blue absorption results were classified as non-irritant, showing absorption values similar to the negative control. These results stay in line with previous reports about RLZ cytotoxicity [16]. Irritation response is the main adverse effect of current topical treatments; therefore, irritation probability and drug release are fundamental aspects to be improved in future management strategies [58].

The angiogenic study showed promising results of RLZ-NLCs demonstrating significant differences against bFGF. Although some controversy is still on the table regarding the angiogenic properties of RLZ [59,60,61], RLZ-NLCs are able to provide values similar to healthy membranes avoiding angiogenesis or any irritation phenomena.

Moreover, the inhibition of proliferation relies on the ability of RLZ to block glutamate release. Aberrant glutamate signaling is known to be involved in survival and proliferation of a wide variety of malignancies [62]. Here, we show the anti-proliferative effects of RLZ in keratinocyte cells promoting cell death. Multiple RLZ mechanisms of action, from promotion of cell cycle arrest to apoptosis sensitization, including autophagy, among others, have been proposed to be beneficial to treat numerous cancer types [11], but other conditions involving hyperproliferation of cells might benefit from its actions. In this sense, RLZ-NLCs would allow for a long-lasting slow release of RLZ suitable to treat uncontrolled proliferation of skin cells in pathologies such as psoriasis, skin dermatoses, actinic keratosis and others. Recently, topical nanocarrier delivery has been proposed as the best choice for psoriasis treatments, due to their named benefits, allowing a prolonged drug liberation and, thus, prolonged action without compromising the compliance of patients or generating the so-called side effects [55,63].

## 4. Materials and Methods

### 4.1. Materials

RLZ was obtained from Thermo Fisher Scientific (Pittsburgh, PA, USA). Lutrol^®^ F68 (LUT), beeswax, peppermint oil, lavender oil and Tween^®^ 80 were obtained from Sigma Aldrich. All other chemical reagents and components used in this research were of analytical grade. A Millipore Milli-Q Plus system was used to obtain purified water.

### 4.2. Preparation of RLZ-NLCs

RLZ-NLCs were prepared using the high-pressure hot homogenization method (HPH) [64]. Briefly, two phases were prepared: a lipid phase containing RLZ, solid lipid (Beeswax) and liquid lipids (mixture of 50% lavender and 50% mint oil); and an aqueous phase, which included the surfactant. The aqueous phase was added to the lipid phase and a pre-emulsion was formed by using the Ultra-Turrax T25 (IKA, Staufen, Germany) at 11.400 rpm for 30 s. Then the emulsion was homogenized using the HPH at 80 °C and 800 bars (Homogenizer FPG 12800, Stanted, UK). Three homogenization cycles were applied and RLZ-NLCs were left overnight in the fridge (4 °C) and physicochemical properties were examined after 24 h.

### 4.3. Physicochemical Characterization of RLZ-NLCs

Z_av_ and PI of RLZ-NLCs were measured using Zetasizer Nano ZS (Malvern Instruments, Malvern, UK) by 1:10 dilution. ZP was estimated by electrophoretic mobility in the same instrument (1:20 dilution was employed) [16]. Measurement conditions were 25 °C and samples were analyzed by triplicate.

EE was determined indirectly [65]. RLZ-NLCs were diluted in water (1:100 dilution) and a filtration centrifugation process was carried out at 14,000 rpm for 15 min (Ultra-0,5 Centrifugal Units MWCO-10000Daltons, EMD Millipore^TM^, Amicon^TM^). The supernatant was collected and the high-performance liquid chromatography (HPLC) technique was used out to measure the free RLZ. Briefly, samples were quantified using the HPLC Waters 2695 (Waters, MA, USA) separation module and a Kromasil^®^ C18 column with a mobile phase formed by water:methanol (30:70). The *EE (%)* was calculated using the following Equation (1) [65]:

(1) $$EE\left(\%\right)=\left(\frac{{RLZ}_{0}(mg)-{RLZ}_{a}(mg)}{{RLZ}_{0}(mg)}\right)\times 100$$

where *RLZ*_0_ refers to the initial *RLZ* weighted and *RLZ*_a_ is the *RLZ* obtained on the supernatant.

### 4.4. Optimitzation of RLZ-NLCs

A systematic design of experiment (DoE) method was employed to optimize the formulation variables of RLZ-NLCs (Table 5), reducing the number of experiments. A four-factor, five-level design was developed consisting of 26 runs, using Statgraphics Centurion^®^ Version 19.3.03.

### 4.5. Interaction Studies of Optimized RLZ-NLCs

#### 4.5.1. Differential Scanning Calorimetry (DSC)

DSC was used to evaluate the thermal profile of RLZ-NLCs as well as their components [66]. A Mettler TA 4000 system (Greinfensee, Switzerland) DSC 25 cell was used and the analysis was carried out by weighting the sample (Mettler M3 Microbalance) and placing it in a perforated aluminum pan. A temperature ramp was applied (25 °C–175 °C, at a rate of 10 °C/min) and data was obtained by using Mettler STARe V9.01dB software (Barcelona, Spain) [16].

#### 4.5.2. Fourier-Transform Infrared Spectroscopy

FTIR spectra of RLZ-NLCs and their components were obtained by Thermo Scientific Nicolet iZ10 equipment, using a ATS diamond and DTGS detector [67].

#### 4.5.3. X-ray Diffraction

XRD was carried out with the aim of analyzing the crystalline and amorphous state of RLZ-NLCs and their components. Samples were disposed between two polyester films and then exposed to CuK radiation (45 kV, 40 mA, = 1.5418 Å) at an angle of 2θ, from 2° to 60°step size and a time of 200 s for step measurement [16].

### 4.6. In Vitro Release Profile Study

A direct dialysis technique was performed to study the RLZ release profile from RLZ-NLCs against free RLZ. Free RLZ was prepared by solving RLZ in 5% *w*/*v* Tween^®^80 at pH 7.4). 8 mL of either free RLZ or RLZ-NLCs were added into a dialysis bag (cellulose membrane, 12–14 kDa, size 3.20/32′ of diameter, Iberlabo). Release media was formed by 180 mL of 5% Tween 80^®^ at pH 7.4 and dialysis bags were placed and left to stir during 24 h at 37 °C. At pre-established timepoints, 300 μL of dialysis medium were extracted and then replaced with release media. RLZ was quantified by HPLC and data were adjusted to the most common pharmacokinetic models.

### 4.7. Stability Assessment

RLZ-NLCs physical stability was measured using Turbiscan^®^ Lab Expert to analyze the light backscattering profile. Therefore, 10 mL of RLZ-NLCs were analyzed at different temperatures (4 °C, 25 °C and 37 °C). Samples were scanned once a month every hour for 24 h [68]. Moreover, Z_av_, PDI, ZP and EE were also measured monthly.

### 4.8. In Vitro Ocular Irritation Assay

#### 4.8.1. HET-CAM

The Hen’s Egg Test Chorioallantoic Membrane (HET-CAM) assay was carried out to study the ocular tolerance of the optimized RLZ-NLCs formulation. For this test, 300 μL of optimized RLZ-NLCs and free RLZ solved with Tween^®^80 at 5% were applied on the chorioallantoic membrane (CAM) of a fertilized egg of 10 days of incubation from the farm G.A.L.L.S.A, Tarragona, Spain. After 5 min of the application, coagulation, vasoconstriction and hemorrhage were evaluated by direct observation. The assay was performed by triplicate, using as a positive control a solution of 0.1 M NaOH, and saline serum (NaCl 0.9 %) as a negative control. The ocular irritation index (OII) was evaluated according to Equation (2):

(2) $$OII=\frac{(301-H)\times 5}{300}+\frac{(301-V)\times 7}{300}+\frac{(301-C)\times 9}{300}$$

where H is hemorrhage, V is vasoconstriction and C is coagulation [69]. Moreover, irritant potential was classified as specified in Table 6 [70].

#### 4.8.2. CAM-TBS

The Chorioallantoic Membrane Trypan Blue Staining (CAM-TBS) assay was carried out to quantitatively evaluate the damage of RLZ-NLCs to the CAM avoiding observers’ subjectivity. For this, 300 µL of the samples were applied to the CAM and after 5 min the CAM was stained for 1 min with 1 mL trypan blue (0.1 % TB in PBS, pH 7.4) as an absorption indicator of dead cells [71]. Afterwards, the CAM was rinsed with distilled water for 20 s, cut off and weighed. The CAM was submerged into 5 mL of formamide for 5 min and absorbance was read at 595 nm [72]. TBS was determined by developing a calibration curve of five concentrations measured by triplicate (5·10^−7^ M, 10^−6^ M, 10^−5^ M, 5·10^−5^ and 10^−4^ M). The OII obtained in the in vitro assay can be categorized according to the amount of TBS absorbed (nmol/mg) as non-irritant/weakly irritant <0.10, moderately irritant 0.10–0.15 or severely irritant >0.15 [73]. All the measurements were carried out by triplicate.

### 4.9. Angiogenesis Study

The anti-angiogenic effect of RLZ and RLZ-NLCs was assessed in vitro using fertilized chicken eggs [74]. For this study, eggs were incubated for 3 days at 37 °C and 85% humidity, and on the 3rd day a hole was opened on the side of the shell to inoculate the samples. Eggs were left 24 h to stabilize, after which 40 µL of sample were inoculated. Membranes were subsequently sealed with sterile transparent tape. After 24 h of stabilization, 40 µL of the formulation were inoculated, images were recorded using a stereomicroscope (STEMI DV4 model, from ZEISS) and the membrane was hermetically sealed. After 48 h images were recorded and 4 % paraformaldehyde was added to the CAM and left for 24 h at 4 °C. After this time, the CAM was extracted and images were recorded for quantification purposes [75]. Subsequently, the density of CAM vessels were measured automatically using ImageJ program as described elsewhere [76].

### 4.10. Cell Culture and Survival/Proliferation

The human keratinocyte HaCaT cell line was grown in DMEM containing 10% FBS. To assess the effect of RLZ-NLCs in keratinocyte proliferation, 0.015 × 10^6^ cells were plated on 96-well plates; one day after, cells were stimulated with different concentrations of RLZ and RLZ-NLCs. Two days after, cell growth was monitored by the reduction of the AlamarBlue reagent (Thermo Fisher) 4 h after its addition as a read out for survival/proliferation. Tecan Magellan fluorescence spectrophotometer was used with 560/590 nm (excitation/emission) filter settings. Clonogenic assays were performed as follows: 200 cells per well were seeded on 6-well plates, and treated with the indicated doses of RLZ, RLZ-NLC, empty NLC or vehicle in duplicate. Ten days after treatment, colonies were stained with Crystal violet for twenty minutes, and after two rinses with water let to dry overnight. Colonies were counted and used to calculate the surviving fraction of cells in each condition.

### 4.11. Protein Lysates and Western Blot

Cells were treated with RLZ or RLZ-NLCs and after 48h cells were lysed with lysis buffer containing 1% Nonidet p-40, 1% deoxycholate, 0.1% SDS, 50 mmol/L HEPES pH 7.5 and 150 mmol/L NaCl, together with protease and phosphatase inhibitors (10 μg/mL aprotinin, 10 μg/mL leupeptin, 86 μg/mL iodoacetamide, and 1 mM PMSF, 1 mM Na_3_VO_4_) [77]. Lysates were boiled in Laemmli buffer, resolved by SDS-PAGE and transferred to a PVDF membrane (Trans-Blot Turbo Transfer Packs, Biorad, Hercules, CA, USA). Membranes were blocked for 1 h at room temperature with 5% skimmed milk and incubated with anti-cleaved PARP (Cell Signaling, Danvers, MA, USA), anti-PCNA (Merck, Rahway, NJ, USA) and anti-βActin (Sigma-Aldrich, Burlington, MA, USA). Signal of secondary HRP-linked antibodies (anti-mouse IgG; Southern Biotech, Birmingham, AL, USA) was detected by enhanced chemiluminescence (ECL, Millipore, Burlington, MA, USA).

## 5. Conclusions

In the present study, a new formulation based on NLCs loading RLZ has been prepared, optimized and characterized with the purpose of overcoming the needs and challenges for topical therapies, such slow release and increased stability. Therefore, the DoE approach was used to optimize RLZ-NLCs containing active liquid lipids and physicochemical properties, and drug interactions were analyzed. RLZ-NLCs showed a suitable stability and demonstrated a good ocular tolerance. In addition, RLZ-NLCs exhibited a sustained release profile during the first 24 h. Of note, RLZ-NLCs proved to possess anti-proliferative effects in keratinocytes. In conclusion, a novel formulation encapsulating RLZ with active liquid lipids has been developed demonstrating the potential to be used against hyperproliferative skin conditions such as psoriasis and atopic dermatitis.

## Figures and Tables

**Table 1:** Obtained results from DoE carried out for RLZ-NLCs optimization.

Independent Variables									Dependent Variables			
	RLZ		Lipid Phase		SL/LL		LUT		Z_AV_ ± SD (nm)	PI ± SD	ZP ± SD (mV)	EE ± SD (%)
	Coded Level	%	Coded Level	%	Coded Level	%	Coded Level	%				
Factorial points												
F1	−1	0.075	−1	4	1	85	1	3.8	112.9 ± 0.6	0.234 ± 0.008	−35.1 ± 1.4	56.7 ± 1.2
F2	+1	0.175	−1	4	1	85	−1	1.4	157.9 ± 0.7	0.157 ± 0.009	−31.5 ± 0.4	74.3 ± 2.4
F3	−1	0.075	1	8	−1	85	1	3.8	160.0 ± 1.2	0.180 ± 0.036	−28.3 ± 0.6	64.1 ± 4.1
F4	+1	0.175	−1	4	−1	65	1	3.8	102.0 ± 0.6	0.189 ± 0.010	−22.5 ± 1.2	78.7 ± 5.7
F5	+1	0.175	1	8	−1	65	−1	1.4	208.5 ± 3.0	0.129 ± 0.008	−28.3 ± 0.4	82.4 ± 4.0
F6	−1	0.075	−1	4	−1	65	−1	1.4	163.6 ± 2.7	0.147 ± 0.014	−25,2 ± 0.7	65.5 ± 7.8
F7	+1	0.175	1	8	1	85	−1	1.4	228.2 ± 1.7	0.141 ± 0.019	−26.8 ± 0.1	82.4 ± 6.5
F8	−1	0.075	1	8	1	85	−1	1.4	219.9 ± 4.7	0.165 ± 0.019	−28.2 ± 0.1	66.3 ± 6.7
F9	+1	0.175	−1	4	−1	65	−1	1.4	158.0 ± 1.7	0.134 ± 0.005	−21.8 ± 0.2	82.2 ± 8.6
F10	−1	0.075	−1	4	−1	65	1	3.8	94,16 ± 1.6	0.185 ± 0.002	−18.2 ± 0.6	64.9 ± 1.1
F11	−1	0.075	−1	4	1	85	−1	1.4	165.2 ± 3.5	0.175 ± 0.031	−33.6 ± 0.7	65.7 ± 9.0
F12	−1	0.075	1	8	−1	65	−1	1.4	214.3 ± 0.8	0.148 ± 0.007	−27.0 ± 0.6	65.8 ± 9.8
F13	+1	0.175	1	8	1	85	1	3.8	163.7 ± 1.0	0.184 ± 0.008	−25.3 ± 1.0	82.7 ± 1.7
F14	+1	0.175	1	8	−1	65	1	3.8	141.9 ± 1.4	0.182 ± 0.011	−20.8 ± 0.6	82.1 ± 3.0
F15	+1	0.175	−1	4	1	85	1	3.8	106.1 ± 1.4	0.214 ± 0.004	−22.0 ± 0.643	81.3 ± 4.1
F16	−1	0.075	1	8	−1	65	1	3.8	164.5 ± 2.7	0.147 ± 0.004	−20.3 ± 0.493	65.3 ± 1.1
Axial points												
F17	0	0.125	2	10	0	75	0	2.6	204.9 ± 1.2	0.174 ± 0.013	−26.7 ± 0.6	77.9 ± 6.4
F18	−2	0.025	0	6	0	75	0	2.6	147.6 ± 0.4	0.164 ± 0.018	−28.5 ± 0.2	0
F19	0	0.125	0	6	0	75	2	5	124.8 ± 3.2	0.186 ± 0.023	−19.0 ± 0.7	75.1 ± 7.6
F20	0	0.125	−2	2	0	75	0	2.6	84.23 ± 0.3	0.185 ± 0.007	−27.7 ± 1.1	73.7 ± 1.5
F21	0	0.125	0	6	−2	55	0	2.6	157.8 ± 3.2	0.125 ± 0.009	−20.6 ± 0.5	73.8 ± 1.3
F22	0	0.125	0	6	0	75	−2	0.2	354.9 ± 7.2	0.184 ± 0.008	−29.6 ± 0.7	71.7 ± 5,4
F23	2	0.225	0	6	0	75	0	2.6	147.8 ± 2.3	0.157 ± 0.029	−22.7 ± 0.1	85.5 ± 7.1
F24	0	0.125	0	6	2	95	0	2.6	173.7 ± 1.7	0.184 ± 0.020	−31.6 ± 0.4	74.7 ± 1.1
Central points												
F25	0	0.125	0	6	0	75	0	2.6	158.2 ± 2.4	0.181 ± 0.015	−30.6 ± 0.8	76.6 ± 9.7
F26	0	0.125	0	6	0	75	0	2.6	152.6 ± 3.0	0.153 ± 0.004	−29.2 ± 2.5	74.9 ± 1.8

**Table 2:** Optimized formulation and physicochemical properties of RLZ-NLCs.

[RLZ] (%)	[Lipid Phase] (%)	[SL/LL] (%)	[LUT] (%)	Z_av_ ± SD (nm)	PI ± SD	ZP ± SD (mV)	EE ± SD (%)
0.167	9.68	67	3.03	192.6 ± 0.8	0.161 ± 0.019	−25.3 ± 0.17	87.16 ± 2.1

**Table 3:** Kinetic parameters of mathematical models fitted to the RLZ release of free RLZ and RLZ-NLCs.

Kinetic Model	Parameters	Free RLZ	RLZ-NLC
Korsmeyer-Peppas	K (min^−1^)	0.1589	0.1254
	R^2^	0.9957	0.9934
	*n*	0.8558	0.8161

**Table 4:** Physicochemical properties of RLZ-NLCs after their storage at different temperatures.

Temperature	Day	Z_av_ (nm)	PI	ZP (mV)
4 °C	0	187.70 ± 1.97	0.175 ± 0.013	−22.71 ± 0.61
	30	180.90 ± 0.59	0.166 ± 0.012	−18.40 ± 0.01
	60	179.80 ± 0.78	0.159 ± 0.016	−16.73 ± 0.82
25 °C	0	185.80 ± 2.08	0.155 ± 0.016	−22.15 ± 0.21
	30	178.80 ± 2.25	0.148 ± 0.015	−12.40 ± 0.10
	60	169.70 ± 2.10	0.140 ± 0.010	−10.13 ± 0.60
38 °C	0	177.50 ± 1.71	0.176 ± 0.011	−23.16 ± 0.72
	30	164.70 ± 1.82	0.157 ± 0.012	−9.01 ± 0.01
	60	No data *	No data *	No data *

* nanoparticles were degraded and the count rate was too low to perform a measurement.

**Table 5:** Matrix of the factorial design with coded levels for the optimization of RLZ-NLCs.

Factor (%)	Level 1 −2	Level 2 −1	Level 3 0	Level 4 +1	Level 5 +2
RLZ	0.025	0.075	0.125	0.175	0.225
Lipid Phase	2	4	6	8	10
SL/LL	55	65	75	85	95
LUT	0.2	1.4	2.6	3.8	5

**Table 6:** HET-CAM test product classification according to their ocular irritation index (OII).

Irritation Level	OII
Non-irritating	≤0.9
Weakly Irritating	0.9< OII ≤ 4.9
Moderately irritating	4.9< OII ≤ 8.9
Irritating	8.9< OII ≤ 21

## Data Availability

Not applicable.

## Supplementary Materials

The following supporting information can be downloaded at: https://www.mdpi.com/article/10.3390/ijms24098053/s1.
