# Supplementary figures and images for: Riluzole-Loaded Nanostructured Lipid Carriers for Hyperproliferative Skin Diseases
Source: Int J Mol Sci. 2023 Apr 29;24(9):8053. doi: 10.3390/ijms24098053 (PMC10179084; doi:10.3390/ijms24098053)

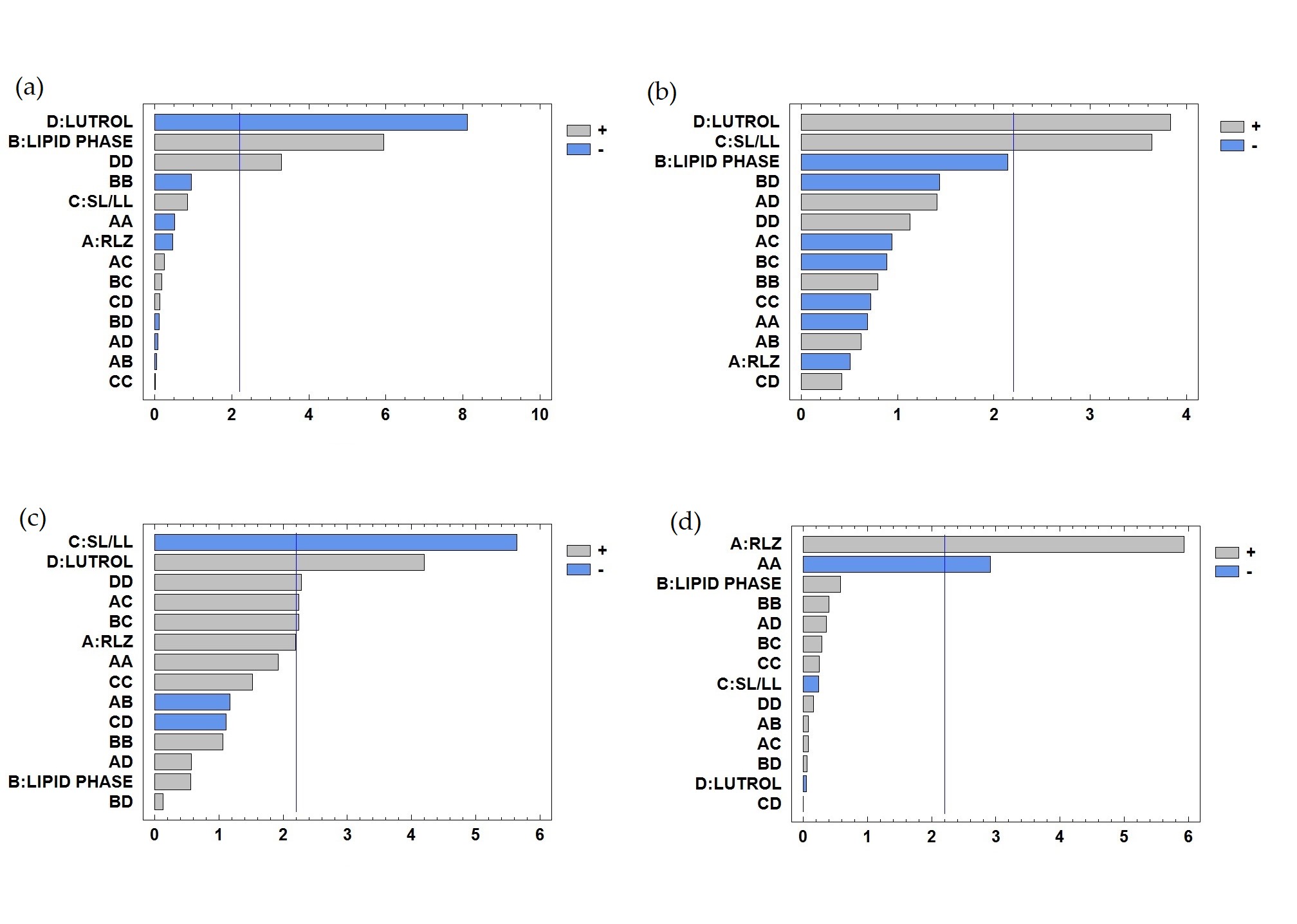

Supplement: Supplementary file 1 [file ijms-24-08053-s001.zip › ijms-2337992-supplementary.jpg]
